# *BARD1* nonsense variant c.1921C>T in a patient with recurrent breast cancer

**DOI:** 10.1002/ccr3.793

**Published:** 2017-01-04

**Authors:** Jennifer Gass, Madeline Tatro, Patrick Blackburn, Stephanie Hines, Paldeep S. Atwal

**Affiliations:** ^1^ Center for Individualized MedicineMayo Clinic4500 San Pablo Road SouthJacksonvilleFlorida32224USA; ^2^ Department of MedicineDivision of Diagnostic & Consultative MedicineMayo Clinic4500 San Pablo Road SouthJacksonvilleFlorida32224USA; ^3^ Department of Clinical GenomicsMayo Clinic4500 San Pablo Road SouthJacksonvilleFlorida32224USA

**Keywords:** *BARD1*, *BRCA1*, breast cancer, genetic testing, tumor suppressor

## Abstract

One of the strongest risk factors predisposing patients to breast cancer is a positive family history. In our study, we describe a patient diagnosed with multiple breast cancer tumors. Genetic analysis revealed a pathogenic variant in *BARD1*, which is associated with an increased risk of developing certain types of cancer.

## Introduction

Breast cancer is the most common form of cancer among women. The majority of these cases are sporadic; however, hereditary risk factors are reported in 30% of patients 1, 2. Through linkage analysis, candidate gene sequencing, and genomewide association studies (GWAS), many of the familial risk factors have been identified and categorized as high, moderate, and low risk, based on their probability of causing disease 3. Variants in high‐risk genes, such as *BRCA1* and *BRCA2,* greatly increase the susceptibility of developing breast and ovarian cancer. Additional studies have discovered several moderate‐ and low‐risk genes that also lead to the development of breast cancer 4. Clinical genetic testing is frequently used to pinpoint these causes. Understanding the origins of disease can assist clinicians in the diagnosis, prognosis, and recommendation of therapy for these patients. In our case study, we describe a patient with a positive family history of cancer who had several recurrences of breast cancer over the last 30 years of her life. Through genetic panel screening, we discovered a deleterious variant in the tumor suppressor, BRCA1‐associated RING domain 1 (*BARD1*).

## Case History

In this case, we present a 76‐year‐old female with a history of multiple bilateral breast cancers. Initially, she was diagnosed with stage I invasive ductal carcinoma of the left breast and underwent a lumpectomy plus axillary lymph dissection at the age of 45. All lymph nodes were negative. During postsurgery, she received adjuvant radiation. Twenty years later, at 65 years of age, she was diagnosed with stage 0 ductal carcinoma in situ (DCIS) and received a left mastectomy. Within a year, she had returned with a swollen right axillary lymph node and was then diagnosed with stage II breast cancer. She then received a right lumpectomy with right axillary lymph node dissection. A pathological evaluation of the removed tumor revealed it to be an invasive ductal carcinoma grade 3, negative for ER, PR, and HER2. Furthermore, she had one positive lymph node out of six. After surgery, she received adjuvant dose‐dense chemotherapy for approximately 5 months. This treatment included four cycles of Adriamycin followed by two cycles of Taxol, two cycles of Abraxane, and finally four cycles of Cytoxan. Subsequently, she also received adjuvant‐like radiation therapy to the right breast and regional lymphatics. It was previously recommended that she undergo *BRCA1/BRCA2* variant analysis given her personal history of breast cancer, which was negative. In late 2011, at the age of 72, she was diagnosed with stage 1A right breast cancer which included a 0.5‐cm invasive ductal carcinoma grade 1 and an associated 0.8‐cm DCIS. Further testing discovered the carcinoma to be ER positive (over 50%), PR positive (11–50%), and HER‐2 negative by FISH with a 1.13 ratio but positive by immunohistochemistry. The patient underwent a right mastectomy plus sentinel lymph node biopsy. Lympho‐vascular invasion and surgical margins were negative, and she began adjuvant endocrine therapy with letrozole.

**Figure 1:**
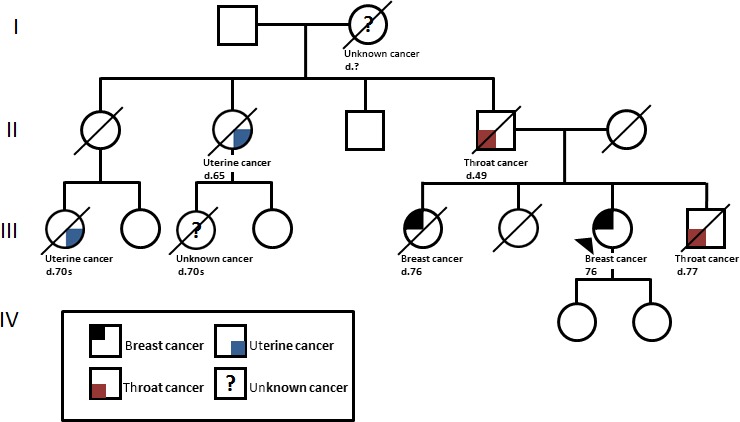
Multiple cancer family pedigree. The proband (III‐7) was diagnosed with multiple breast cancers. Genetic testing revealed a novel *BARD1* variant. Several cancers were also reported in various family members. Each affected family member is labeled by cancer and present age or age at death.

Moreover, our patient describe an extensive family history of cancer, including a sister who died from breast cancer at 76, a paternal aunt who died from uterine cancer at 65, and a paternal first cousin who died of uterine cancer in her 70s (Fig. 1). Her paternal first cousin and grandmother also had cancer, site unknown. Her father and brother, who were both long‐time smokers, both died of throat cancer, aged 49 and 77, respectively.

## Diagnosis and Investigations

Based on her recurring bouts of breast cancer and the widespread family history of numerous cancers, she was referred to the Department of Clinical Genomics to undergo genetic testing. The Breast/Ovarian Cancer Panel from GeneDx was recommended and revealed a potentially pathogenic c.1921C>T variant (p.Arg641Ter) in *BARD1* (MIM*601593). This panel sequences 21 different genes associated with hereditary breast and ovarian cancer. (http://www.genedx.com/test-catalog/available-tests/breastovarian-cancer-panel/) Cancer risk associations of *BARD1* are not fully understood, although several studies have suggested that variants in this gene may be linked to an increased risk of developing breast and ovarian cancers 5, 6, 7, 8.

## Discussion

A family history of breast cancer is one of the greatest risk factors for disease. To date, there are over 30 different genes linked to an increased risk of developing breast cancer 1, 3, 9, 10. The majority of familial cases are caused by high‐penetrance genes, such as *BRCA1, BRCA2, PTEN,* and *TP53*. Other risk factor variants can be difficult to classify as familial due to reduced penetrance, increased frequency within controls, and lack of molecular characterization. Therefore, it is essential to report case studies of patient's with variants in these newer, moderate and lower risk genes.

In our patient, we identified a deleterious variant in *BARD1* denoted c.1921C>T. This substitution introduced a premature stop codon and is predicted to cause loss of normal protein function through either truncation or nonsense‐mediated mRNA decay. Genomic screenings of large disease cohorts have reported this variant in triple negative breast cancer, neuroblastoma, and pancreatic cancer 11, 12, 13, 14, 15. Our study is the first to provide a detailed description of patient history, diagnosis, and treatment in a patient harboring this particular variant, which is considered pathogenic.

BARD1 was first discovered as a binding partner to BRCA1 using a yeast two‐hybrid screening 16. In fact, BARD1 and BRCA1 contain many structural similarities, including an N‐terminal RING finger domain and two C‐terminal BRCT domains. N‐terminal binding of BARD1 to BRCA1 forms a heterodimer complex which stabilizes both proteins and acts as an E3 ubiquitin ligase 17. Furthermore, the association of BARD1 and BRCA1 induces nuclear localization, DNA repair, and tumor suppression 18. In addition, BARD1 performs several BRCA1‐independent functions, which include inhibition of polyadenylation of mRNA during DNA damage as well as inducing apoptosis when it interacts with p53 19, 20, 21. As BARD1 is involved in genomic stability, disruptions of this protein due to genetic variants often lead to the development of various cancers 11, 22. Therefore, genetic testing of *BARD1* in addition to translational research is necessary to diagnose and determine potential therapeutic options for patients with pathogenic variants.

## Conclusions

Due to the increased probability and recurrence of breast cancer in patients with *BARD1* pathogenic variants, early and routine breast cancer screening such as yearly mammograms and breast MRIs is recommended. Our patient has had four episodes of breast cancer throughout her life starting at the age of 45, with only 1 year between the second and third diagnoses, revealing the importance of screening these patients. Genetic testing and counseling are also suggested for her adult children, who have a 50% chance of inheriting this variant and passing it on to their offspring. As our patient had an increased rate of cancer within her extended family, it would have been of great interest to test these members to determine whether other cancers (i.e., uterine and throat) are also associated with *BARD1* variants. However, she is the only living member still alive. Thankfully, routine screenings and follow‐up examinations can help prevent the spread or mortality associated with familial breast cancer.

## Ethics Approval and Consent

Diagnosis, treatment, and counseling were performed following the principles of medical ethics. The authors have obtained proper consent to publish data collected from this patient. All forms have been properly signed and are available upon request.

## Authorship

JG: involved in organization and wrote the first draft. MT: prepared manuscript. PB: reviewed and critiqued. SH: involved in project execution, reviewed, and critiqued. PA: involved in project execution, reviewed, and critiqued.

## Conflicts of Interest

There are no conflict of interests to report. All authors have approved the content of the manuscript.
